# Supplementary material for: Electronic photoreceptors enable prosthetic visual acuity matching the natural resolution in rats
Source: Nat Commun. 2022 Nov 4;13:6627. doi: 10.1038/s41467-022-34353-y (PMC9636145; doi:10.1038/s41467-022-34353-y)
Supplement: Supplementary file 3 — Reporting Summary [file 41467_2022_34353_MOESM3_ESM.pdf]

nature portfolio

Corresponding author(s): Bing-Yi Wang

Last updated by author(s): Sep 16 2022

Reporting Summary

Nature Portfolio wishes to improve the reproducibility of the work that we publish. This form provides structure for consistency and transparency in reporting. For further information on Nature Portfolio policies, see our Editorial Policies and the Editorial Policy Checklist.

Statistics

For all statistical analyses, confirm that the following items are present in the figure legend, table legend, main text, or Methods section.

n/a	Confirmed	
☐	☒	The exact sample size (*n*) for each experimental group/condition, given as a discrete number and unit of measurement
☐	☒	A statement on whether measurements were taken from distinct samples or whether the same sample was measured repeatedly
☐	☒	The statistical test(s) used AND whether they are one- or two-sided<br>*Only common tests should be described solely by name; describe more complex techniques in the Methods section.*
☐	☒	A description of all covariates tested
☐	☒	A description of any assumptions or corrections, such as tests of normality and adjustment for multiple comparisons
☐	☒	A full description of the statistical parameters including central tendency (e.g. means) or other basic estimates (e.g. regression coefficient) AND variation (e.g. standard deviation) or associated estimates of uncertainty (e.g. confidence intervals)
☐	☒	For null hypothesis testing, the test statistic (e.g. *F*, *t*, *r*) with confidence intervals, effect sizes, degrees of freedom and *P* value noted<br>*Give P values as exact values whenever suitable.*
☒	☐	For Bayesian analysis, information on the choice of priors and Markov chain Monte Carlo settings
☒	☐	For hierarchical and complex designs, identification of the appropriate level for tests and full reporting of outcomes
☒	☐	Estimates of effect sizes (e.g. Cohen's *d*, Pearson's *r*), indicating how they were calculated

Our web collection on statistics for biologists contains articles on many of the points above.

Software and code

Policy information about availability of computer code

Data collection	Espion E3 system (Diagnosys LLC)
Data analysis	Custom code in MATLAB (vR2017a and vR2020a); custom code in Python 3.8.8; custom model in COMSOL Multiphysics 5.6

For manuscripts utilizing custom algorithms or software that are central to the research but not yet described in published literature, software must be made available to editors and reviewers. We strongly encourage code deposition in a community repository (e.g. GitHub). See the Nature Portfolio guidelines for submitting code & software for further information.

Data

Policy information about availability of data

All manuscripts must include a data availability statement. This statement should provide the following information, where applicable:

- Accession codes, unique identifiers, or web links for publicly available datasets
- A description of any restrictions on data availability
- For clinical datasets or third party data, please ensure that the statement adheres to our policy

Source data are provided with this paper. The raw in-vivo VEP data and in-vitro data that support the findings of this study are available from the corresponding author upon reasonable request.

Human research participants

Policy information about studies involving human research participants and Sex and Gender in Research.

Reporting on sex and gender	N/A
Population characteristics	N/A
Recruitment	N/A
Ethics oversight	N/A

Note that full information on the approval of the study protocol must also be provided in the manuscript.

Field-specific reporting

Please select the one below that is the best fit for your research. If you are not sure, read the appropriate sections before making your selection.

☒ Life sciences ☐ Behavioural & social sciences ☐ Ecological, evolutionary & environmental sciences

For a reference copy of the document with all sections, see nature.com/documents/nr-reporting-summary-flat.pdf

Life sciences study design

All studies must disclose on these points even when the disclosure is negative.

Sample size	9 RCS rats and 6 LE rats were included in this study. The sample size was determined to achieve sufficient statistical significance with the least number of distinct individuals, based on past experimental evidences.
Data exclusions	No data were excluded from the analysis.
Replication	We have measured each independent sample multiple times to ensure the reproducibility of stated findings.
Randomization	The animals participating this study were randomly selected from our colony. The choice of implanted device type was also randomized.
Blinding	Blinding was not relevant nor possible in this study. Researchers can visualize the implanted devices during surgery and measurements. However, no human biases were introduced into the analysis and statistical study due to a priori knowledge of implant type in each animal.

Reporting for specific materials, systems and methods

We require information from authors about some types of materials, experimental systems and methods used in many studies. Here, indicate whether each material, system or method listed is relevant to your study. If you are not sure if a list item applies to your research, read the appropriate section before selecting a response.

Materials & experimental systems

n/a	Involved in the study	
☒	☐	Antibodies
☒	☐	Eukaryotic cell lines
☒	☐	Palaeontology and archaeology
☐	☒	Animals and other organisms
☒	☐	Clinical data
☒	☐	Dual use research of concern

Methods

n/a	Involved in the study	
☒	☐	ChIP-seq
☒	☐	Flow cytometry
☒	☐	MRI-based neuroimaging

Animals and other research organisms

Policy information about studies involving animals; ARRIVE guidelines recommended for reporting animal research, and Sex and Gender in Research

Laboratory animals	Rats: RCS (Royal College of Surgeons) and Long Evans, male and female, 6 to 9 months old

Wild animals	No wild animals were used in this study.
Reporting on sex	Findings are dependent upon animal sex.
Field-collected samples	No field-collected samples were used in this study.
Ethics oversight	Stanford Administrative Panel on Laboratory Animal Care

Note that full information on the approval of the study protocol must also be provided in the manuscript.
